# Learning from Graduate and Undergraduate Public Health Virtual Internship Experiences with State Title V Agencies During COVID-19, Summer 2020

**DOI:** 10.1007/s10995-021-03278-1

**Published:** 2021-11-18

**Authors:** Arden Handler, Rebecca Greenleaf, Christine T. Bozlak, Victoria Moerchen, Kris Pizur-Barnekow, Cindy San Miguel, Olivia Sappenfield, Gabriella Masini

**Affiliations:** 1 Community Health Sciences, Center of Excellence in Maternal and Child Health, University of Illinois at Chicago School of Public Health, 1603 W. Taylor, Chicago, IL 60612 USA; 2 National MCH Workforce Development Center, University of North Carolina at Chapel Hill Gillings School of Global Public Health, 412C Rosenau Hall, CB# 7445, Chapel Hill, NC 27599-7445 USA; 3 Health Policy, Management, and Behavior, Maternal and Child Health Program, University at Albany School of Public Health, One University Place, Rm 173, Rensselaer, NY 12144 USA; 4 College of Health Sciences, University of Wisconsin-Milwaukee, PO Box 413, Milwaukee, WI 53201-0413 USA; 5 Department of Rehabilitation Sciences & Technology, College of Health Sciences, University of Wisconsin-Milwaukee, 979 Enderis Hall, PO Box 413, Milwaukee, WI 53201 USA; 6 Sinai Urban Health Institute, 1111 West 16th Street, Chicago, IL 60608 USA; 7 Epidemiology-Biostatistics, University of Illinois at Chicago School of Public Health, 1603 W Taylor St., Chicago, IL 60612 USA; 8 Center of Excellence in MCH, University of Illinois at Chicago School of Public Health, 1603 W Taylor St, Chicago, IL 60612 USA

**Keywords:** Maternal and Child Health, Graduate and undergraduate students, Virtual internships, State and territorial Title V agencies

## Abstract

**Background:**

Since summer 2014, the National MCH Workforce Development Center has placed students from MCH public health graduate (Centers of Excellence and Catalyst) and undergraduate (MCH Pipeline) programs, all funded by the Maternal and Child Health Bureau, in summer internships with state and territorial Title V agencies. In 2020, due to the COVID-19 pandemic the Title V MCH Internship Program was offered virtually.

**Participants and Methods:**

This manuscript includes quantitative and qualitative data from 2017 to 2020 generated by both Title V MCH Internship student interns (n = 76) and their preceptors (n = 40) with a focus on a comparison between the 2020 virtual year and the 2017–2019 years.

**Results:**

Evaluation data from the 2017 to 2020 Title V MCH Internship Program from both students and preceptors revealed the implementation of a robust and successful internship program in which students increased their confidence in a variety of team, mentorship, and leadership skills while gaining direct exposure to the daily work of state Title V agencies. However, students and preceptors identified more challenges during 2020 compared to previous years.

**Conclusions:**

The COVID-19 Pandemic was both a disruption and a catalyst for change in education. While there were clearly some challenges with the pivot to a virtual Title V MCH Internship Program in summer 2020, students were able to participate in meaningful internship experiences. This success can be attributed to the ability of the internship sponsor to engage in best practices, including extensive planning and provision of ongoing support to the students. Going forward, it is recognized that virtual internships may facilitate access to agencies in distant locales, eliminating issues related to housing and transportation. When both virtual and in-person relationships are available, those responsible for internship programs, including the Title V MCH Internship, will need to weigh these type of benefits against the potential missed opportunities students may have when not able to participate in on-site experiences.

## Significance

*What is already known on this subject?* The literature on virtual internships for public health students is nascent. What has been published to date, documents both the challenges of virtual internships in general as well as outlines best practices for ensuring success when public health internships are virtual.

*What does this study add?* This is the first paper describing the implementation of a virtual internship program for MCH graduate public health and undergraduate MCH Pipeline students. The success of this virtual internship experience, which has typically paired students with state and territorial Title V agencies in person, provides evidence that this approach may be fruitful in the future. Virtual Title V internships may be particularly useful for working with Title V states agencies in small state capitols with housing and transportation challenges as well as for territorial Title V agencies which may be thousands of miles from students’ universities or home base.

## Introduction

From the enactment in 1935 of Title V of the Social Security Act, the education and training of the maternal and child health (MCH) workforce has been viewed as essential to enabling Title V and its partners to address the multiple challenges facing women, children, and families (Kavanagh, 2015). The Division of MCH Workforce Development (DMCHWD) within the Maternal and Child Health Bureau (MCHB) “partners with state MCH programs, academic institutions, professional organizations, and other health training programs of the federal government to ensure that MCH workforce development programs are grounded in emerging and evidence-based practices” (DMCHWD Fact Sheet. MCHB, 2019). The multiple training and education investments of MCHB include the Centers of Excellence in MCH (CoEs-MCH) and since 2015, the MCH Public Health Catalyst Programs (Catalysts), both of which provide graduate level training in MCH in Schools of Public Health. In addition, since 2006, MCHB supports undergraduate MCH Pipeline Programs (now called Leadership, Education, and Advancement in Undergraduate Pathways Training Program or LEAP) which expose economically and educationally disadvantaged students including those from under-represented groups to MCH professions.

Since 2013, MCHB has supported the National MCH Workforce Development Center (NMCHWDC) at the University of North Carolina at Chapel Hill, Gillings School of Global Public Health (UNC-SPH) to provide support to states in a changing health care environment. One of the inaugural programs of the NMCHWDC was the *Title V MCH Internship Program*, administered by MCH faculty and staff from the University of Illinois at Chicago School of Public Health (UIC-SPH). Formerly called the *Paired Practica Program,* this internship pairs teams of students from CoE, Catalyst, and Pipeline Programs with state Title V agencies (Handler et al., 2018) to: (1) provide both graduate and undergraduate MCH students with exposure to state Title V agencies; (2) assist state Title V agencies with the implementation of a variety of projects related to health care and MCH Block Grant transformation; and, (3) develop the mentorship skills of MCH graduate students while exposing MCH undergraduates to “mentors” close in age and experience. The creation of student teams was designed to pair students from larger, more developed MCH programs (CoEs-MCH) with students in smaller graduate MCH programs (Catalysts) in Schools of Public Health, and with students from undergraduate MCH Pipeline Programs.

During the academic year of 2019–2020, plans were underway for in-person internships at 12 state and territorial health agencies for summer 2020. However, when the nation “shut down” in March 2020 due to the COVID-19 pandemic, the decision was made to switch the Summer 2020 Title V MCH Internship to a virtual experience. To add to the nascent literature on virtual public health internships, this manuscript presents the results of the experiences of interns and preceptors participating in the Title V MCH Internship from 2017 to 2020, with emphasis on comparing the *2020 virtual year with the 2017–2019 in-person years*. Lessons learned and suggestions for best practices for virtual internships for MCH undergraduate and graduate public health students working in state Title V agencies and beyond are described.

## Methods

This manuscript includes quantitative survey and qualitative data from 2017 to 2020 generated by both Title V MCH Internship student interns and preceptors with a focus on comparison between the 2020 virtual year and the 2017–2019 in-person years. The experience of the first three years (summers 2014–2016) of this internship program have previously been described (Handler et al., 2018). Likewise, data from the summer 2021 virtual summer were not available at the time of the writing of this manuscript.

### Student Interns

Student interns participated in both pre (prior to the internship) and post-surveys (after the completion of the internship) each year. The pre-internship survey included questions that measured student interns’ expectations of their own skills, their confidence with respect to working in teams and with senior (or junior) individuals, and their future career and academic plans. The post-survey included similar questions in order to measure the internship’s impact and to provide an opportunity for qualitative feedback.

In the analysis and tables included in the results below, responses are categorized into the most positive response (e.g., highly likely, highly confident, great extent), the second most positive response (e.g., likely, confident, moderate amount), and other (e.g., the remaining less favorable responses). We present information on student interns’ characteristics, student confidence working with peers and junior/senior individuals, their confidence in teamwork skills, and their future career plans. Results are reported by year. Due to small sample sizes, CoE-MCH and MCH Catalyst responses are typically combined into an overall graduate student intern response; significance tests were not conducted.

### Preceptors

After the completion of the internship each summer, preceptors from the participating state and territorial Title V agencies responded to a survey that evaluated student interns’ performance and contribution. In the analysis and table presented below, preceptor responses are categorized into the most positive response (e.g., highly likely, highly confident, great extent) and other less favorable responses. We present preceptor perceptions of the skills and experiences of the student interns, their overall assessment of student interns’ performance, and their likelihood of recommending the Title V MCH Internship to other Title V programs. Results are reported by year.

### Comparison Between Virtual Year (2020) and Prior Years (2017–2019)

Four of the authors (AH, RG, VM, CSM) reviewed the quantitative data to determine if responses to each question in the 2020 year appeared to have increased/decreased or stayed the same as previous years. There were two approaches to this determination. If the data were available at the post-survey only, in order to be counted as an increase or an improvement, the response to each question in 2020 needed to be more positive (higher percent) than the response to that question in *all* previous years (2017–2019). To be considered as a decrease, the response to the question in 2020 needed to be less positive (lower percent) than in *all* previous years. The 2020 year responses were determined to be the “same” as the scores in previous years if the scores in previous years were both higher and lower or the same as the 2020 year scores. If pre and post-survey data were both available, we examined whether the change from pre to post was in the same direction (also noting which direction) for the 2020 year versus all previous years. For most questions, we examine the graduate experience separately from the undergraduate experience.

### Qualitative Analysis

Two forms of qualitative data contribute to telling the story of students and preceptors participating in the Title V MCH Internship in 2017–2019 and 2020:

1. *Thematic analysis of student and preceptor open-ended post-survey comments overall and related to participating in a virtual internship*. Three authors (KB, CB, RG) reviewed these comments in an iterative fashion and documented student and preceptor concerns and successes with participation in a virtual internship. Each author viewed the prior assessments of the other authors, and all reviewing authors then came to agreement on how to categorize the comments. The student comments were not stratified by graduate/undergraduate status. In addition, student comments on the pre-survey about their concerns and fears with respect to participating in a virtual internship were documented.
2. *Thematic analysis of students’ comments provided during the Title V MCH Internship virtual presentations which took place at the end of the internship (late July) in 2020*. The presentations were delivered via ZOOM to internship staff and the staff of the state Title V agencies. Two authors (CB and KB) analyzed the presentations in which students described their engagement with the projects, challenges, accomplishments, and future steps. Presentations ranged from 6 to 16 min in length. Each author reviewed 6 of the 12 video presentations and captured key quotes and reflections using the following categories: state, project focus, key quotes, and overarching theme. The two authors then met to review and develop consensus regarding the themes. These themes were not stratified by graduate/undergraduate status.

This study was not considered to be human subjects research under federal regulations by the University of North Carolina IRB Office of Human Research Ethics and as such, did not require IRB approval.

## Results

**Fig. 1:**
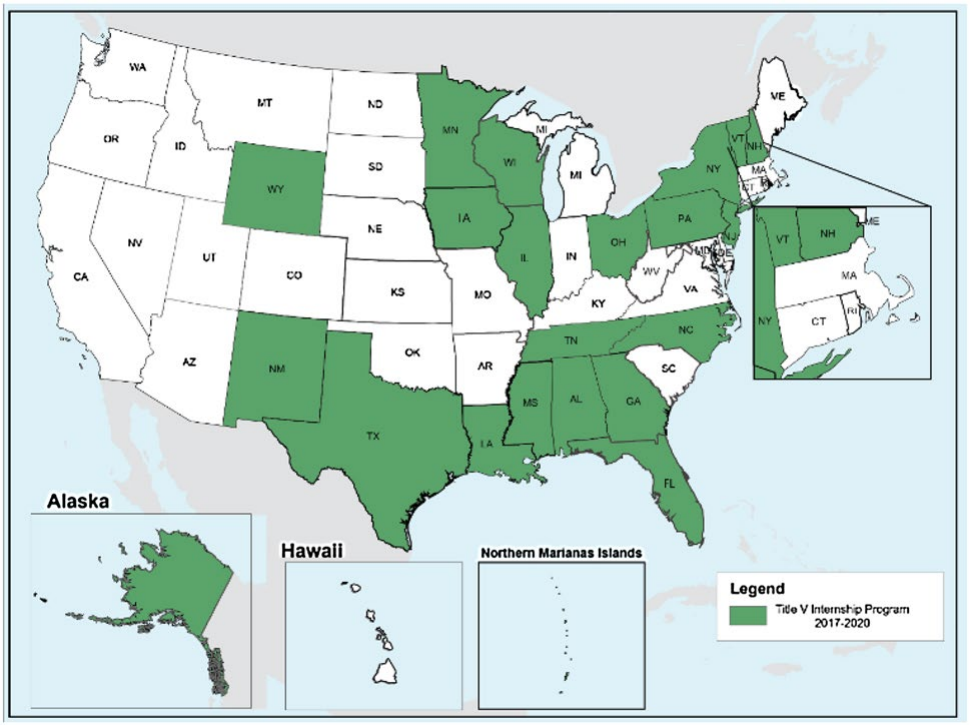
Map of Title V MCH Internship Program participating states and territories 2017–2020

From 2017 to 2020, the Title V MCH Internship Program placed trainees from 11 of the 13 Centers of Excellence, all 5 of the MCH Public Health Catalyst Programs, and 5 of the 6 MCH Pipeline Programs funded during that period (Table 1) in internships in 22 states and one territory (see Fig. 1). All students received a $4500 stipend for participating in the internship.

**Table 1:** Title V MCH Internship Summer 2017–2020 participating MCH programs

CoE-MCH programs	MCH Catalyst programs	MCH Pipeline programs
Boston University	Drexel University	Alabama State University
Emory University	Florida International University	Baylor College of Medicine
Harvard University	Rutgers University	Kennedy Krieger Institute
Johns Hopkins University	Texas A&M University	University of South Florida
Tulane University	University at Albany—State University of New York	University of Wisconsin-Milwaukee
University of Alabama at Birmingham		
University of California, Berkeley		
University of Illinois-Chicago		
University of Minnesota		
University of North Carolina at Chapel Hill		
University of Washington

### Comparison of Virtual Year 2020 to Three Previous Years

#### Quantitative Analysis: Student Results

From 2017 to 2020, 79 student interns participated in the Title V MCH Internship Program, and 76 completed pre- and post-internship surveys about their experience. Of the 76 student interns, the majority participated in a CoE-MCH and MCH Catalyst team (n = 26) or in a CoE-MCH and MCH Pipeline team (n = 29). Sixty-two (82%) of the 76 student interns were graduate students. Over the four years, 55% of the graduate students were non-Hispanic white, 24% were non-Hispanic Black, 13% were Hispanic, and 5% were non-Hispanic Asian. Among undergraduates, 36% were non-Hispanic Black, 36% were non-Hispanic Asian, and 21% were Hispanic. While 42% of graduate students were from underserved populations, 71% of undergraduates were from underserved populations (Data Not Shown).

The student evaluation survey asks graduate students about their confidence in working with peers and junior individuals and asks undergraduate students about their confidence in working with senior individuals (post-survey data only available; Data Not Shown). In 2020, there appeared to be less confidence among graduate students on the post-survey in working in teams with peers, connecting a peer with others in their own MCH networks, connecting a junior person with others in their own MCH network, and sharing their public health journey with a junior person compared to all previous years. However, confidence in working in teams with junior individuals and imparting skills to a junior person were both the same in the 2020 virtual year as in previous years. Interestingly, there appeared to be greater confidence in imparting knowledge and skills to a peer during the 2020 virtual internship compared to previous years. For undergraduate students, based on the post-survey, the virtual internship did not appear to differ from previous years (typically, undergraduate confidence was lower than graduate students’ confidence across all years for these questions) with respect to their confidence in working with senior individuals, seeking out individuals who expose students to new aspects of public health/MCH, and engaging with senior leaders in the field of public health/MCH. The one area where there appeared to be less confidence at the post-survey for undergraduate students was in asking a senior person for clarification about an idea or activity.

Students were also asked about their confidence with respect to teamwork skills. In the 2020 virtual year as was typically true in all previous years, graduate students’ confidence in *all teamwork skills* increased/improved from the pre to post-survey (Table 2). These skills included being able to articulate a shared vision, setting and meeting goals/deadlines, seeking out and using resources relevant to a project, learning from others in a team environment, actively listening to others and encouraging contributions from everyone, and functioning effectively as an informal or formal MCH leader. The picture looks different for undergraduate students (Table 3). For most attributes, the undergraduates appeared less confident at the 2020 post-survey than at the pre-survey compared to previous years, often moving from a highly confident stance at the pre-survey in 2020 to just a confident response. This is in contrast to previous years in which undergraduates often moved from confident to highly confident in their responses related to functioning in a team environment.

**Table 2:** Graduate student interns’ confidence in teamwork skills (percent), 2017–2020 (n = 62)

	2017 (n = 13)		2018 (n = 14)		2019 (n = 16)		2020 (n = 19)	
	Pre	Post	Pre	Post	Pre	Post	Pre	Post
Developing and articulating a shared vision, roles, and responsibilities								
Highly confident	53.9	69.2	50.0	64.3	18.8	37.5	21.1	42.1
Confident	46.1	30.8	42.9	35.7	62.5	62.5	68.4	52.6
Other	0.0	0.0	7.1	0.0	18.8	0.0	10.5	5.3
Setting and meeting goals/deadlines^1^								
Highly confident	53.8	69.2	64.3	82.2	46.9	71.9	50.0	79.0
Confident	46.2	30.8	32.2	17.9	50.1	28.2	47.4	21.1
Other	0.0	0.0	3.6	0.0	3.2	0.0	2.7	0.0
Seeking out and using resources relevant to a project								
Highly confident	53.9	92.3	64.3	57.1	18.8	75.0	26.3	63.2
Confident	46.1	7.7	21.4	35.7	68.8	25.0	47.4	36.8
Other	0.0	0.0	14.3	7.1	12.5	0.0	26.3	0.0
Taking the initiative to address a project issue								
Highly confident	38.5	92.3	57.1	71.4	25.0	68.8	26.3	57.9
Confident	53.9	7.7	28.6	28.6	50.0	31.3	52.6	42.1
Other	7.7	0.0	14.3	0.0	25.0	0.0	21.1	0.0
Learning from others in a team environment								
Highly confident	69.2	92.3	78.6	92.9	50.0	62.5	52.6	79.0
Confident	30.8	7.7	21.4	7.1	50.0	37.5	47.4	21.1
Other	0.0	0.0	0.0	0.0	0.0	0.0	0.0	0.0
Providing opinions and feedback when working in a group								
Highly confident	46.2	84.6	35.7	50.0	18.8	75.0	42.1	57.9
Confident	46.2	15.4	57.1	50.0	62.5	25.0	36.8	42.1
Other	7.7	0.0	7.1	0.0	18.8	0.0	21.1	0.0
Actively listening to others and encouraging contributions from everyone								
Highly confident	69.2	92.3	64.3	85.7	43.8	68.8	52.6	73.7
Confident	23.1	7.7	35.7	14.3	50.0	31.3	47.4	26.3
Other	7.7	0.0	0.0	0.0	6.3	0.0	0.0	0.0
Functioning effectively as an informal or formal MCH leader								
Highly confident	30.8	76.9	50.0	42.9	31.3	62.5	26.3	52.6
Confident	15.4	23.1	28.6	57.1	37.5	37.5	26.3	47.4
Other	53.9	0.0	21.4	0.0	31.3	0.0	47.4	0.0
Contributing to improvements in MCH population health								
Highly confident	23.1	76.9	50.0	50.0	31.3	62.5	15.8	57.9
Confident	38.5	23.1	28.6	50.0	43.8	37.5	42.1	42.1
Other	38.5	0.0	21.4	0.0	25.0	0.0	42.1	0.0

^1^Setting and meeting goals was asked as two separate questions in 2018 and 2020 so the percentages reflect average responses at the given level for those years

**Table 3:** Undergraduate student interns’ confidence in teamwork skills (percent), 2017–2020 (n = 14)

	2017 (n = 2)		2018 (n = 3)		2019 (n = 6)		2020 (n = 3)	
	Pre	Post	Pre	Post	Pre	Post	Pre	Post
Developing and articulating a shared vision, roles, and responsibilities								
Highly confident	0.0	100.0	66.7	66.7	33.3	83.3	0.0	0.0
Confident	100.0	0.0	33.3	33.3	66.7	16.7	33.3	66.7
Other	0.0	0.0	0.0	0.0	0.0	0.0	66.7	33.3
Setting and meeting goals/deadlines^1^								
Highly confident	0.0	50.0	50.0	83.4	33.3	75.0	0.0	0.0
Confident	100.0	50.0	33.3	16.7	58.4	25.0	100.0	100.0
Other	0.0	0.0	16.7	0.0	8.4	0.0	0.0	0.0
Seeking out and using resources relevant to a project								
Highly confident	0.0	100.0	0.0	33.3	16.7	83.3	33.3	0.0
Confident	50.0	0.0	100.0	66.7	33.3	16.7	66.7	66.7
Other	50.0	0.0	0.0	0.0	50.0	0.0	0.0	33.3
Taking the initiative to address a project issue								
Highly confident	50.0	50.0	33.3	0.0	33.3	83.3	0.0	0.0
Confident	50.0	50.0	66.7	100.0	50.0	16.7	100.0	66.7
Other	0.0	0.0	0.0	0.0	16.7	0.0	0.0	33.3
Learning from others in a team environment								
Highly confident	50.0	100.0	66.7	33.3	50.0	100.0	66.7	0.0
Confident	50.0	0.0	33.3	66.7	50.0	0.0	33.3	100.0
Other	0.0	0.0	0.0	0.0	0.0	0.0	0.0	0.0
Providing opinions and feedback when working in a group								
Highly confident	0.0	50.0	33.3	33.3	33.3	83.3	33.3	0.0
Confident	50.0	50.0	66.7	66.7	33.3	16.7	33.3	33.3
Other	50.0	0.0	0.0	0.0	33.3	0.0	33.3	66.7
Actively listening to others and encouraging contributions from everyone								
Highly confident	100.0	100.0	66.7	66.7	50.0	83.3	66.7	0.0
Confident	0.0	0.0	33.3	33.3	33.3	16.7	33.3	100.0
Other	0.0	0.0	0.0	0.0	16.7	0.0	0.0	0.0
Functioning effectively as an informal or formal MCH leader								
Highly confident	0.0	50.0	0.0	0.0	16.7	66.7	33.3	0.0
Confident	50.0	50.0	66.7	100.0	33.3	16.7	33.3	66.7
Other	50.0	0.0	33.3	0.0	50.0	16.7	33.3	33.3
Contributing to improvements in MCH population health								
Highly confident	0.0	50.0	0.0	0.0	66.7	83.3	33.3	0.0
Confident	50.0	50.0	66.7	66.7	16.7	16.7	33.3	100.0
Other	50.0	0.0	33.3	33.3	16.7	0.0	33.3	0.0

^1^Setting and meeting goals was asked as two separate questions in 2018–2020 so percentages reflect average responses at the given level for those years

Internship students were also asked whether participation in the Title V MCH internship affected their career goals and their plans after graduation with respect to participation in the MCH workforce or MCH education (Data Not Shown). With respect to the extent to which participation in the Title V MCH Internship affected interns’ career goals, the responses for graduate students in 2020 were on par with previous responses (very much: 50% in 2018, 31.3% in 2019, and 47.4% in 2020; no data available in 2017). This was also true for their after graduation plans. In each year, 2017–2020, graduate students at the post-survey reported a greater likelihood of seeking a job in a Title V program in a state health agency, in a community based or other organization that focuses on MCH, and of seeking additional education in MCH after the completion of the internship than prior to the internship. The only two exceptions to this phenomenon occurred in 2018, when graduate students were equally likely to state they might seek a job in a Title V agency and less likely to report seeking additional education in MCH after the internship than before the internship. The 2020 responses of the undergraduate interns regarding the effect of the Title V MCH Internship on their career plans varied from previous years (33% responding very much in 2018 and 2019, and 0–66.7% responding moderately in 2018 and 2019 respectively, compared to 0% responding very much and 33.3% responding moderately in 2020). However, the reflections of the undergraduate interns on the effect of this internship on their plans after graduation were quite consistent with previous years, with most somewhat likely or highly likely to seek MCH related jobs or additional education in MCH.

#### Quantitative Analysis: Preceptor Results

In each year, almost all preceptors responded to the evaluation survey (post-survey only) provided at the end of the internship (10/13 in 2017; 8/9 in 2018; 10/12 in 2019; 12/12 in 2020). From 2018 through 2020, preceptors responded that their student teams performed well overall (Data Not Shown). The proportion of preceptors who felt their student intern teams performed very well was lower in 2017 compared to the later 3 years. More specifically, all or nearly all preceptors from 2018 through 2020 reported: (1) their graduate student intern was able to mentor the undergraduate student intern to a great extent; (2) that the interns were matched very well to the site and project; (3) the students’ work was very useful to their agency; and, (4) the students contributed a great deal to a project that helped their agency respond to the changing health care environment. However in the 2020 virtual year, preceptors were less likely to agree that the students were matched very well as a team and were less likely to agree that the students were very successful in completing their tasks than in 2018 and 2019. Slightly fewer preceptors in 2020 found the interns’ work very useful to the agency compared to 2018 and 2019. On the other hand, preceptors were more likely in 2020 than in any previous year to report that students contributed to a project that helped the agency increase its focus on a systems approach to MCH services.

Preceptor assessment of student intern performance by year is included in Table 4. As shown in Table 4, in the 2020 virtual year, slightly fewer preceptors reported that the students were likely to a great extent to set and meet goals than in 2018 and 2019, and only 50% reported that the students were familiar to a great extent with the working of state Title V programs (same as in 2017). Conversely, 100% of preceptors in 2020 reported student teams successfully sought out resources relevant to the project, took initiative to address issues that arose, worked effectively with their student partner to accomplish project goals, and successfully worked with other agency staff as needed to a great extent. Lastly, 50% of preceptors in 2017, all preceptors in 2018, 90% of preceptors in 2019, and 83% of preceptors in 2020 were very likely to recommend the Title V MCH Internship Program to other local and state health care agencies (Data Not Shown).

**Table 4:** Preceptor overall assessment of student intern performance (percent) (n = 40)

	2017 (n = 10)	2018 (n = 8)	2019 (n = 10)	2020 (n = 12)
Set and met goals/deadlines^1^				
Great extent	25.0	100.0	90.0	87.5
Other	75.0	0.0	0.0	12.5
Successfully sought out resources relevant to the project				
Great extent	50.0	100.0	100.0	100.0
Other	50.0	0.0	0.0	0.0
Took initiative to address issues that arose				
Great extent	25.0	100.0	100.0	100.0
Other	75.0	0.0	0.0	0.0
Worked effectively with their student partner to accomplish project goals				
Great extent	100.0	87.5	100.0	100.0
Other	0.0	12.5	0.0	0.0
Familiar with the working of state Title V programs				
Great extent	50.0	75.0	70.0	50.0
Other	50.0	25.0	30.0	50.0
Familiar with the changing health care environment				
Great extent	50.0	62.5	70.0	58.3
Other	50.0	37.5	30.0	41.7
Successfully worked with other agency staff as needed				
Great extent	100.0	100.0	100.0	100.0
Other	0.0	0.0	0.0	0.0

^1^Preceptors were asked about setting goals/deadlines and meeting goals/deadlines as two separate questions in 2020

#### Qualitative Exploration

Prior to the start of summer 2020, students expressed their concerns about participating in a virtual internship on the pre-survey. These included fears related to getting to know their student partner, receiving timely feedback from their student partner and preceptor, personal time management, connectivity and technology issues, gaining access to agency documents and information, and just general anxiety related to the COVID-19 pandemic. The students’ and preceptors’ comments on their post-internship surveys validated some of their initial concerns about a virtual internship and aligned with the comments expressed by the students in the public presentations (see below). While students expressed some concern that they did not reap the full benefits of the internship (“I just felt like if it wasn’t virtual, I would have been exposed to more hands on stuff in the department of public health”), the students also had great praise for their preceptors (“My preceptors were amazing. I think they supported me to the fullest extent and provided as much opportunity as they could have for exposure and networking within MCH”). The preceptors also described several challenges: not being able to just drop in and check on students' progress, the inability of the students to meet staff pulled into COVID-19 activities, the lack of access to state datasets and confidential files because the students were not onsite, and issues related to coordinating meeting times across time zones. On the other hand, preceptors felt that the virtual environment meant that: “new and innovative ways to connect with public health staff and communities had to be developed and implemented”. The preceptors mentioned ways in which they overcame some of the challenges including: “having a predetermined work plan and utilizing the initial meeting with the intern to review, discuss, and make adjustments”, and, “we were able to collaborate so well on Microsoft Teams, and even able to help fellow public health colleagues learn how to use it! (The students are so tech savvy)”.

Based on their virtual celebration presentations held in July 2020, the student interns indicated they experienced multiple challenges during the summer 2020 virtual internship including difficulties in the ability to complete their initial deliverables, as exemplified by the following: “Timeline was a challenge—we were on a strict timeline…only 8 weeks”. Another student stated: “Due to COVID, I was not able to pilot test the focus group interview guide”. Several of the students mentioned that their preceptors and departments were busy responding to COVID-19. However, many of the interns experienced a “silver lining” as they were able to incorporate experiences related to the pandemic into their deliverables for their internships, as exemplified by the following: “I was able to incorporate COVID into interviews”; and, “We were able to make (social media) content on MCH and COVID that felt timely and important”. In addition, students were grateful that they were able to still have a summer 2020 internship: “A lot of people were not able to pursue internships this summer” (due to the pandemic) and (we) were very grateful to have the opportunity”. Although the pandemic and virtual nature of the internships created some issues with student interns feeling sufficiently “integrated” into their assigned state agencies, many of the student interns provided praise for their preceptors’ efforts despite the difficult circumstances, as exemplified in the following: “It was amazing to meet so many MCH professionals and learn what they do”… “We learned so much about the branches of the state (health) department and how they work to reach a similar goal”. Another team of students reflected: “Remote work poses challenges with connecting and feeling integrated with staff. Thankfully, our preceptors were really awesome at scheduling weekly group and individual check-ins with each of us. We also held video check-ins when we had complex tasks that we wanted to discuss”.

Interestingly, based on comments during the celebration presentations, the virtual internship appeared to enhance MCH leadership competencies (MCHB, 2018) related to *self* (reflective, be aware of self in respect to others) and *others* (communication) as the student interns’ solutions to their frustrations with the virtual internship included comments about communicating frequently, setting up a calendar to meet with their preceptor, stakeholders and others, and being mindful/respectful of the strain/stress that the pandemic created for self and others.

Finally, several of the interns expressed gratitude to the NMCHWDC Pipeline Team for their support during the internship. One intern explicitly stated that the internship impacted their future career aspirations: “I think I’ll definitely consider working in state health departments and elsewhere—maybe the CDC.”

## Discussion

In the summer of 2020, the Title V MCH internship, which had operated for six previous years in person, switched to a virtual internship, allowing the students an opportunity to fulfill their internship requirements while meeting the needs of multiple state Title V agencies. This was not the case for many other students across the US (and internationally). In fact, during 2020, many students who had planned summer internships were unable to fulfill these plans due to the COVID-19 pandemic shutdown. One large state university survey of students reported a 40% loss of job offers or internships (Aucejo et al., 2020).

Overall evaluation data from the 2017 to 2020 Title V MCH Internship Program collected from both students and preceptors reveal the implementation of a robust and successful internship program in which students gained a variety of skills and increased their confidence in a variety of team, mentorship, and leadership abilities while gaining direct exposure to the daily work of state Title V agencies. The rapid transition to a virtual internship in the summer 2020 Title V MCH Internship Program was possible, in part, because our existing internship model already was hybrid in its construction. In other words, although the interns in past summers were in-person at the state agencies, they participated in a nationwide leadership and mentoring program which connected them remotely to the other interns placed at agencies across the nation, and to the learning and support provided by the internship leadership across several universities. We were fortunate that our model also had several program elements in place that aligned with best practices for distance education and virtual internships (Kachra & Brown, 2020; Roddy et al., 2017; Werner & Jeske, 2021): well-developed state/agency projects carefully developed by the internship staff in conjunction with the state preceptors; synchronous engagement of interns with the internship faculty and each other before, during and after the internship; identification and continuous monitoring of learning goals; celebration of learning and project outcomes; expectations of and support for collaborative and proactive communication; and, perhaps most significantly, connecting student interns within and across state projects.

It is important to note that the virtual Title V MCH internship experience in summer of 2020 was not a previously planned experience but was the result of a pivot during a global pandemic in which students’ and preceptors’ work and personal lives were upended in multiple ways. In addition, the summer of 2020 was a time of racial reckoning in the US after the repeated murders of black persons and other persons of color by the police including those of George Floyd, Ahmaud Arbery, Brianna Taylor, Daunte Wright, Adam Toledo, Anthony Alvarez, Andrew Brown and the untold others who are killed by police on a daily basis. It was also a time when communities of color were hardest hit by COVID and disproportionately affected by COVID-related mortality. Concurrently, many students from less advantaged backgrounds were struggling to stay engaged with their education, while facing challenges in their remote environments. All of these factors provide important contextual information for understanding the results of the comparisons made between the evaluation data from the 2020 and 2017–2019 years and the issues both students and preceptors were confronting while trying to have/provide a successful internship experience.

Importantly, distance or virtual learning, while not completely new to most students was also not the norm for the students participating in this internship pre-pandemic. The 2020 Title V MCH Internship Program engaged both interns and agency preceptors who were mostly novices in the online learning context. In prior research, student anxiety about virtual learning was highly negatively correlated with value of virtual learning and performance (Lederman, 2020). Similarly, in another study, student confidence in learning was related to confidence with remote technology and mentor/instructor confidence and skill in remote teaching (Landrum, 2020). Although we are unable to test the relationship between students’ anxieties and success related to working in a virtual environment during COVID, when comparing the virtual internship experience of 2020 to 2017–2019, the results suggest that while the internship continued to be successful, there were stressors. For example, in 2020, there appeared to be a decrease in confidence among graduate students in a number of areas including working in teams with peers, connecting a peer or junior person with others in their own MCH networks, and sharing their public health journey with a junior person compared to all previous years. Likewise, in the 2020 virtual year, preceptors were less likely to agree that the students were matched very well as a team and were less likely to agree that the students were very successful in completing their tasks than in 2018 and 2019; in addition, slightly fewer preceptors in 2020 found the interns’ work very useful to the agency compared to 2018 and 2019.

Based on these data, it is clear that the 2020 Title V MCH Internship experience was not without challenges. Such challenges have been previously identified in the literature (Goodman, 2015; Hora, 2020; Linkov et al., 2021) including the need for students to be more self-directed and independent and to have excellent communication, organizational, and online project management skills, the distractions of working in a non-work environment (exacerbated during the COVID-19 pandemic), the inability of preceptors to directly “observe” the students, and the inability of students to directly “observe” their preceptors.

Despite the challenges, students and preceptors in the 2020 Title V MCH Internship Program proved to be resilient with most students gaining the teamwork and leadership skills that are the aim of this internship. As stated previously, the successes can be attributed to a number of strategies that align with best practices previously identified in the literature (Goodman, 2015; Linkov et al., 2021). These strategies include extensive planning by the Title V MCH Internship staff built on a structure that existed prior to the pandemic, enabling an easy transition to the virtual space. The Title V MCH Internship Program provided multiple training sessions for the interns before the internship began and throughout the summer focused on working in a virtual environment, working independently, working in teams and with preceptors, and provided direct support to the student interns through regular “team” meetings with a coach and other opportunities for checking in.

As shown in these data, in some cases, MCH undergraduate students from MCH Pipeline Programs appeared to face more challenges benefiting from the Title V MCH Internship Program than graduate students in all years including the 2020 virtual year. However, the undergraduates in the 2020 year appeared less confident at the post-survey than the pre-survey than in previous years with respect to functioning in a team environment. We hypothesize that the undergraduates, who were all from underrepresented population groups and more likely to be from underserved communities, may have been more affected by the uncertainties associated with the pandemic, including challenging family or housing circumstances, the loss of extended family or friends due to COVID-19, and the emotional charge of the events of Spring and Summer of 2020 related to racial injustice.

Undergraduates were also less likely to state that the Title V MCH Internship had a major effect on their career plans in 2020 compared to previous years. On the other hand, most stated that they were somewhat likely or highly likely to seek MCH-related jobs or additional education in MCH, which was consistent with prior years. For undergraduate interns, the temporal distance from completion of their bachelor’s degree (typically at least one year after the internship) plus anticipation of graduate education prior to entering the workforce likely affects their ability to project the impact of the Title V MCH Internship on their future career trajectories. This supports the need for continued engagement of these students in their Pipeline (now known as “LEAP”) programs in the academic year after the completion of the summer internship so that the longer term impact of this experience can be shaped and supported by their MCH faculty mentors at their home universities.

Finally, while this evaluation of the Title V MCH Internship Program’s virtual year experience compared to prior years is limited by small numbers which prevented statistical testing, this is countered by the evaluation’s utilization of both quantitative and qualitative data, the collection of data from both students and preceptors, and the ability to compare students’ experiences over time. Importantly, comparing the responses from the 2020 virtual year to the responses from the previous years was conducted using a consistent approach with agreement on the comparison of all question responses by four of the paper’s authors. In addition, the qualitative comments on the surveys and in the presentations were also reviewed by multiple authors who documented student and preceptor concerns and successes with respect to participation in the 2020 virtual internship in an iterative fashion.

While the Title V Internship does not maintain specific mentoring support once students return to their universities, each year, we support the previous summer’s interns to submit abstracts to the Association of Maternal and Child Health Programs (AMCHP) annual meeting and provide resources for their attendance. In addition, the Title V MCH Internship Program team will be conducting a follow-up survey and interviews with the 2020 cohort in 2023; follow-up contact with interns several years after the completion of the internship is standard practice within the Title V MCH Internship Program.

### Conclusions for Practice

The COVID-19 Pandemic was both a disruption and a catalyst for change in higher education (Lucey & Johnson, 2020). The unexpected shift to virtual education including virtual internships meant that students, their teachers/advisors, and practitioners all had to acquire new skills and to consider new approaches to learning, all of which has opened up new possibilities for the future. While there were clearly some challenges and missed opportunities with the virtual internship program, preceptors and students in the Title V MCH Internship Program appeared resilient and adaptable and were able to participate in meaningful internship experiences. This success can be attributed to the ability of the internship sponsor to engage in best practices including extensive planning and provision of ongoing support to the student interns.

Going forward, the potential benefits of virtual internships for public health students in general, and MCH students in particular, must be considered. Participating in virtual internships with state Title V agencies in particular, eliminates the need to travel to smaller state capitols with housing and transportation challenges especially when time and resources are limited, while still exposing students to the inner workings of state Title V agencies. Likewise, the potential to participate in a virtual internship facilitates engagement in internships with territorial public health agencies that may be thousands of miles from students’ universities or home base. A virtual internship also allows students to potentially continue research or practice based projects at their home university and may allow for additional support from their home institution. Those responsible for internship programs, including the Title V MCH Internship Program, will need to weigh these benefits against the notion that “in-person” internships are better because they provide students with “on the ground” real-world experience. Hybrid internship models in which students participate in person for some of the time followed by virtual participation might provide the best of both approaches.

## References

[CR1] Aucejo EM, French J, Araya M, Zafar B. (2020). The impact of Covid-19 on student experiences and expectations: Evidence from a survey. *National Bureau of Economics Working Paper 27392*. 10.3386/w27392. 10.1016/j.jpubeco.2020.104271 PMC7451187 32873994

[CR2] Division of Maternal and Child Health Workforce Development (DMCHWD). (2019). *Fact Sheet. health resources services administration, maternal & child health*. Retrieved June 24, 2021, from https://mchb.hrsa.gov/training/documents/fs/DMCHWD-Factsheet.pdf

[CR3] Goodman J (2015). Virtual practicums within an MPH program: A career development case study. Health Promotion Practice..

[CR4] Handler A, Klaus J, Long-White D, Roth M, Greenleaf R, Sappenfield O, Cilenti D (2018). Innovations in maternal and child health: Pairing undergraduate and graduate maternal and child health students in summer practica in state title V agencies. Maternal and Child Health Journal..

[CR5] Hora M (2020). What to do about internships in light of the COVID-19 pandemic? A short guide to online internships for colleges, students, and employers.

[CR6] Kachra R, Brown A (2020). The new normal: Medical education during and beyond the COVID-19 pandemic. Canadian Medical Education Journal.

[CR7] Kavanagh L (2015). Challenges and opportunities facing maternal and child health (MCH) professionals. Maternal Child Health Journal.

[CR8] Landrum B (2020). Examining students’ confidence to learn online, self-regulation skills and perceptions of satisfaction and usefulness of online classes. Online Learning Journal.

[CR9] Lederman D (2020). The student view of this spring's shift to remote learning.

[CR10] Linkov F, Khanijahani A, Swanson-Bearman B, Akinci F (2021). Remote internship: Practical approaches to sustaining student internships amid public health epidemics. The Journal of Health Administration Education.

[CR12] Lucey CR, Johnson SC (2020). The transformational effects of COVID-19 on medical education. JAMA.

[CR11] Maternal and Child Health Bureau. (2018). *MCH Leadership Competencies 4.0*. Rockville, MD: US Department of Health and Human Services. Retrieved June 25, 2021, from https://mchb.hrsa.gov/training/documents/MCH_Leadership_Competencies_v4.pdf.

[CR13] Roddy C, Amiet D, Chung J, Holt C, Shaw L, McKenzie S, Garivaldis F (2017). Applying best practice online learning, teaching, and support to intensive online environments: An integrative review. Frontiers in Education.

[CR14] Werner J (2021). Ten simple rules for running and managing virtual internships. PLoS Computational Biology..

